# Letter to the Editor (April 23, 2026) concerning the paper “Factors associated with respiratory symptoms in communities living near to and away from the railway used for coal transportation in La Guajira, Colombia”

**DOI:** 10.13075/ijomeh.1896.02888

**Published:** 2026

**Authors:** Alvaro J. Idrovo

**Affiliations:** Universidad Industrial de Santander, Faculty of Health, School of Medicine, Department of Public Health, Bucaramanga, Colombia

**Keywords:** air quality, respiratory symptoms, Colombia, coal mining, agnotology, environmental epidemiology

Dear Editor,

I have read the article by Amaya-Lara et al. [1] on the association between respiratory symptoms and exposure to dust generated at the largest open-cast coal mine in Latin America, and I have several concerns and comments regarding the study's context, methods, and interpretation of findings. These issues should be considered by readers of the “International Journal of Occupational Medicine and Environmental Health” in order to draw more objective conclusions.

First, the authors do not acknowledge that the study is situated within the context of one of the country's major socio-environmental conflicts. The Wayúu Indigenous people have, for decades, stated that mining activities have caused environmental degradation and pollution, as well as adverse effects on population health. Given this situation, the Constitutional Court of Colombia has already expressed concern about the health risks potentially associated with coal mining (Judgment T-614 of 2019) [2].

Second, although I will not elaborate on exposure measurement, as it is widely recognized that this is a complex and imperfect process in environmental epidemiology, the approach selected by the authors appears methodologically sound as an exploratory strategy. However, the study examines respiratory symptoms that were grouped, for statistical analysis, into a single category encompassing manifestations of chronic bronchitis, asthma, or chronic obstructive pulmonary disease (COPD) [3]. It is noteworthy that the multivariable analysis includes variables such as asthma or asthma attacks, as well as pneumonia or bronchitis, as predictors of this composite outcome of respiratory symptoms. This raises the question of possible overadjustment.

Furthermore, the statistical analysis relies on logistic regression to estimate associations, despite the composite outcome being relatively frequent and without clear verification that the assumptions required for the odds ratio to be an appropriate measure are satisfied [4]. Consequently, the reported associations may be overestimated, potentially exaggerating the effect of asthma or asthma attacks.

In light of these considerations, it is pertinent to question whether the study may inadvertently contribute to obscuring a socio-environmental conflict and an environmental health issue. Is the odds ratio masking the effects of coal dust? In Colombia, conducting research in environmental health is particularly challenging due to the prevalence of socio-environmental conflicts and various manifestations of violence. Therefore, the limited number of studies that are conducted should be based on robust designs that address the concerns raised by affected communities, while avoiding the inadvertent promotion of agnotology [5] in environmental health.

## AI USE

AI was used to check the grammar.
